# From 2D MXenes to 3D Carbides: Transformation of Ti_3_C_2_T_*z*_ Thin Films into TiC_*x*_ Carbide Nanolayers

**DOI:** 10.1021/acs.nanolett.6c00187

**Published:** 2026-04-13

**Authors:** Barak Ratzker, Bar Favelukis, Mathias Krämer, Christoph Freysoldt, Yiftach Kushnir, Asaf Nitsan, Alexander Upcher, Nitzan Maman, Or Messer, Dierk Raabe, Baptiste Gault, Maxim Sokol

**Affiliations:** † 28272 Max Planck Institute for Sustainable Materials, Max-Planck-Str. 1, 40237 Düsseldorf, Germany; ‡ Department of Materials Science and Engineering, 26745 Tel Aviv University, P.O.B 39040, Ramat Aviv 6997801, Israel; § Ilse Katz Institute for Nanoscale Science and Technology, 26732 Ben-Gurion University of the Negev, P.O.B 653, Beer-Sheva 8410501, Israel; ∥ Groupe de Physique des Matériaux, Univ Rouen Normandie, CNRS, INSA Rouen Normandie, UMR 6634, Rouen F-76000, France

**Keywords:** MXene, titanium carbide, spin-coating, spark plasma sintering, reactive bonding, interlayer

## Abstract

MXenes are renowned for their versatile uses in various scientific fields as 2D materials, but they also show potential in structural applications utilizing their high-temperature transformation into transition-metal carbides with unique morphologies. Herein, we developed a method to study this transformation in model systems. As a proof-of-concept, MXene thin films (≤20 nm thickness) were deposited on sapphire and subjected to high temperature (1400 °C) and moderate pressure (18 MPa), transforming them into nanolamellar carbides. Structural and compositional analyses of the interface revealed that the Ti_3_C_2_T_*z*_ transformed into TiC_*x*_O_*y*_ (with ∼13 at% O), while halide surface terminations were removed. A small fraction of Li (∼0.2 at%) was detected within the transformed nanolayer, stabilized by the presence of O in the oxycarbide lattice. Ultimately, this methodology provides a framework for studying the MXene-to-MX transformation under controlled conditions, enabling the rational design of nanocomposites and bonding nanolayers for advanced structural and functional materials.

## Graphical abstract

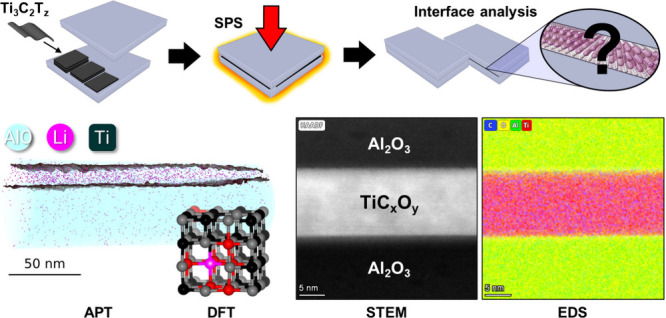

MXenes are 2D transition-metal carbides, nitrides, or carbonitrides, typically synthesized by HF- or molten salt etching of a parent MAX phase metallic A-layer, followed by intercalation (e.g., alkali ions like Li^+^ or Na^+^, or organic molecules), and exfoliation.^1^ During synthesis, MXenes surfaces acquire an abundance of termination groups (e.g., −O, −OH, −F, −Cl) with their chemistry depending on the synthesis process.^2^ Thus, MXenes are denoted by M – transition metal, X – C or N, and T_*z*_ – termination groups, with the most prevalent and widely studied being Ti_3_C_2_T_*z*_. Owing to their exceptional properties, MXenes and MXene-based composites are an increasingly active research subject, spanning a wide range of scientific areas including applications in energy, healthcare, electronics, photonics, tribology, catalysis, sensing, and environmental science.^3–5^

In contrast, MXenes and MXene-based composites are under-represented as structural materials, i.e. where mechanical behavior is the key property of interest. The unique morphologies and processability of MXenes or their bulk lamellar counterparts MX phases offer several benefits when included as reinforcement phases or additives in metal or ceramic composite frameworks.^6^ They can enhance mechanical properties like strength, hardness, toughness, and wear resistance as well as improve densification or particle bonding for powder metallurgy approaches.^6–9^ MXenes are also advantageous regarding processability, as they benefit from high hydrophilicity, colloidal stability, and tunable wettability^10^ – making it easy to mix or deposit them on a variety of materials.

A key feature of this concept is the ability of 2D MXenes to transform into 3D MX phases when heated to high temperatures.^11^ When applying powder metallurgy approaches, the high-temperature sintering process will strongly influence the final state and structure of the material, whether it is 2D (hexagonal) Ti_3_C_2_T_*z*_ or 3D (cubic) TiC_*x*_ reinforcement. For example, rapid low-temperature sintering (<1200 °C) enabled by pressure-assisted densification techniques limits the MX transformation and are able to produce MXene/ceramic composites; as was shown for Ti_3_C_2_T_*z*_-based nanocomposites with oxide matrix materials such as ZnO,^12^ Al_2_O_3_,^7^ and ZrO_2_.^13^ Conversely, processing at high temperatures (>1200 °C) or using extended holding times will facilitate complete transformation of Ti_3_C_2_T_*z*_ into bulk TiC_*x*_.^6,11,14^ TiC_*x*_-based ceramic composites, facilitated by high-temperature Ti_3_C_2_T_*z*_ transformation and/or reactions, have already been demonstrated for a variety of ceramic matrix materials consolidated by spark plasma sintering (SPS), such as Al_2_O_3_,^14^ SiC,^15^ TiC,^8^ and ZrB_2_.^9^

Here, we set out to develop a methodology for studying the high-temperature-driven transformation of 2D Ti_3_C_2_T_*z*_ into its 3D MX counterpart. This was achieved by coating sapphire dies with Ti_3_C_2_T_*z*_ MXene prepared from a pure Ti_3_AlC_2_ precursor (Figure S1), creating a sandwich by placing another sapphire die on top and bonding the laminate structure by heating under applied pressure. This approach provides the means to analyze the carbide product at near-atomic scale and any interfacial reactions following the high-temperature treatment under controlled conditions. Ultimately, the methodology demonstrated herein could be used across nearly any combination of 2D material sandwiched between metal or ceramic materials.

Figure 1a–c illustrate the procedure employed. The first step involves coating one side of a sapphire die (cut from a c-plane(0001) wafer) with a uniform MXene thin film of controlled thickness (Figure 1a). Further details on the spin-coating process and atomic force microscopy characterization of the deposited MXene thin films are detailed elsewhere.^16^ Note that an HCl cleaning step is applied between each deposition cycle,^16^ which cleanses halide salt impurities and also removes intercalated Li ions, shrinking the MXene *d*-spacing from ∼ 1.49 nm to ∼ 1.32 nm (Figure S2). The second step requires using a procedure like SPS to apply uniaxial pressure and heating (Figure 1b), similar to diffusion bonding processes.^17,18^ The bonded sandwich structure can then be cut to enable precise examination of the formed bonding layer cross section (Figure 1c). In the showcased example, sapphire dies were spin-coated with Ti_3_C_2_T_*z*_ films with thicknesses in the ∼ 6–20 nm range, corresponding to 1–3 coating cycles. They were heat treated at 1400 °C together under an applied pressure of 18 MPa in a single SPS process, yielding bonded “sandwich” laminated parts (Figure 1d-g).

**Figure 1:**
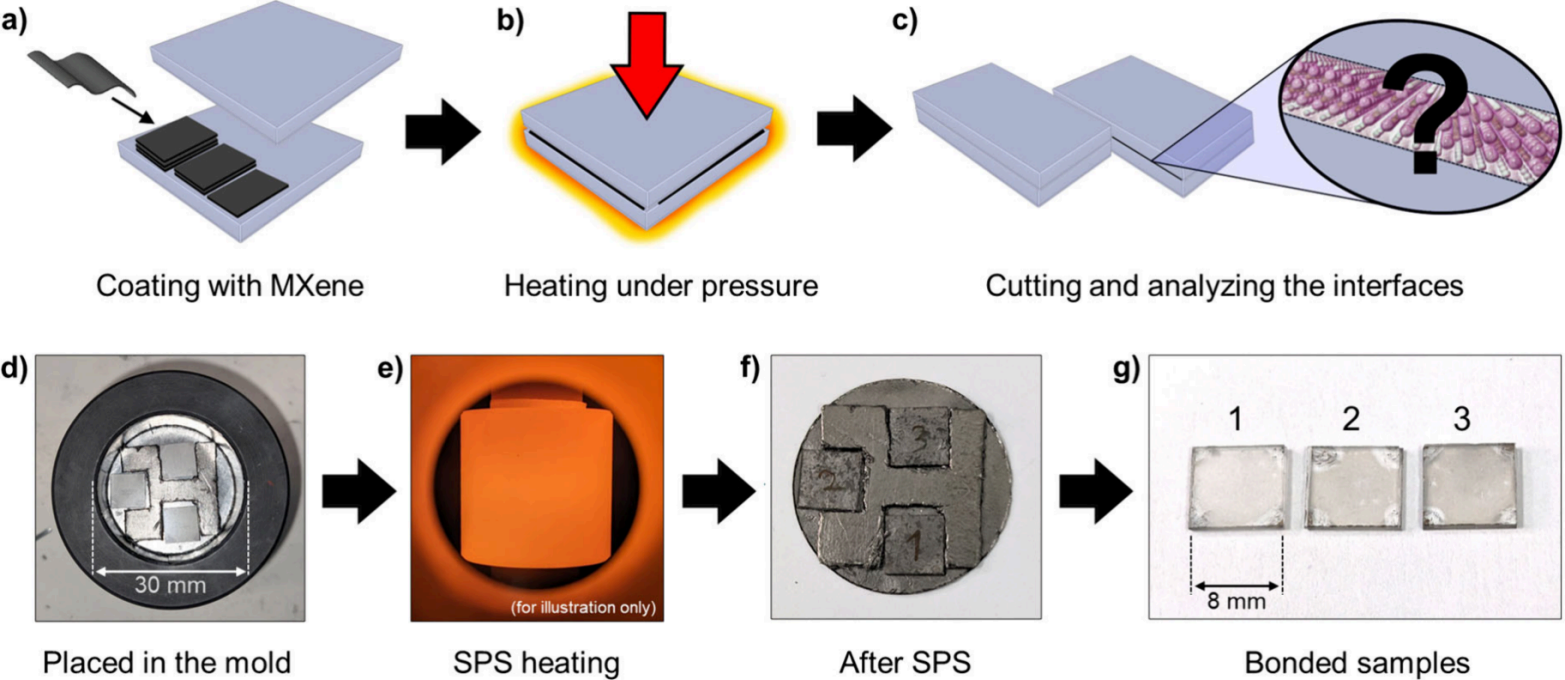
**Procedure of transforming MXene into MX phase under controlled settings.** Illustration of the experimental procedure: (a) coating with the desired thickness of MXene thin film, (b) heating to high temperature while applying external uniaxial pressure, (c) cutting and analysis of the interface nanolayer after MXene-to-MX transformation. A proof-of-concept demonstration: (d) sapphire dies coated with Ti_3_C_2_T_*z*_ thin films of ∼ 6–20 nm thickness arranged together in a 30 mm graphite mold, (e) heating of the graphite mold during SPS (appearance of an exposed mold at 900 °C for illustration purposes), (f) the extracted samples after the SPS process still covered in graphite foil residue, and (g) the bonded samples after being cleaned and polished – numbering (1–3) indicates the amount of MXene spin-coating cycles.

The bonded sapphire remained reasonably transparent (Figure 1g), with the in-line transmittance of ∼ 70–78% at 650 nm (81–90% of the theoretical transmittance of sapphire) decreasing with the thickness of the deposited MXene (Figure S3). No absorbance peaks related to the surface plasmon of Ti_3_C_2_T_*z*_ are observed.^16^ Notably, the nanolayer thickness of the bond means that this process could be applied for joining ceramics where sufficient transparency is a critical requirement. For instance, a transparent window that includes a laminated TiC_*x*_ interlayer may offer a unique combination of hardness and transparency with an active conductive layer^19^ that can also withstand extremely harsh high temperature, radiation, or corrosive environments.

Structural and compositional analyses at the nanoscale were conducted to gain insight into the high-temperature transformation of Ti_3_C_2_T_*z*_ into TiC_*x*_. Scanning electron microscopy (SEM) with focused ion beam (FIB) was used to prepare lamellae for examination of the interfaces by scanning transmission electron microscopy (STEM). Figure 2 presents high-resolution STEM analysis of the 3-coating cycles MXene-derived TiC_*x*_ layer situated between sapphire on either side. The effective bonding between Al_2_O_3_ and TiC can, in part, be attributable to their similar thermal expansion coefficients, i.e., approximately 8 × 10^6^ /°C at 1400 °C.^20^ There was no observable difference in the transformation of MXene to MX, regardless of the initial film thickness. Notably, after the transformation voids made up roughly 25% of the MX layer, estimated by image analysis of the interface (Figure 2b). Otherwise, for the most part, a seamless interface was observed between the carbide layer and the enclosing sapphire (Figure 2c). Further examples of residual voids in the bonding region are shown in Figure S4. It should be noted that for extremely thin coatings (i.e., after 1 spin-coating cycle) there were regions where the sapphire surfaces ended up in direct contact following the diffusion bonding process. This resulted in a more discontinuous MX layer between Al_2_O_3_ regions, formation of infrequent relatively large voids, less intermittent voids, and an overall smaller void fraction (see Figure S4). Nevertheless, the void fraction should be taken as a rough estimation since it is based on the limited TEM observations. In some regions, for the 3-cycle coating, the TiC_*x*_ could span hundreds of nanometers without noticeable voids (Figure S5). Additionally, occasional small-volume interfacial voids between part of the TiC_*x*_ and Al_2_O_3_ could also be found, as shown near the boundary of two TiC_*x*_ nanograins (Figure S6). Lastly, in the 1-cycle-coated samples, some regions showed evidence of an interfacial reaction with the Al_2_O_3_ (Figure S7). As these were only found sporadically in that particular sample (no such regions found in the 3-coating-cycles sample) it is not inherent to the process. We propose that it could be attributed to the presence of localized impurities (e.g., residual fluorides) which have not been adequately removed during the MXene preparation and deposition cleaning process,^16^ as these are expected to be able to react with Al_2_O_3_ at high temperatures.

**Figure 2:**
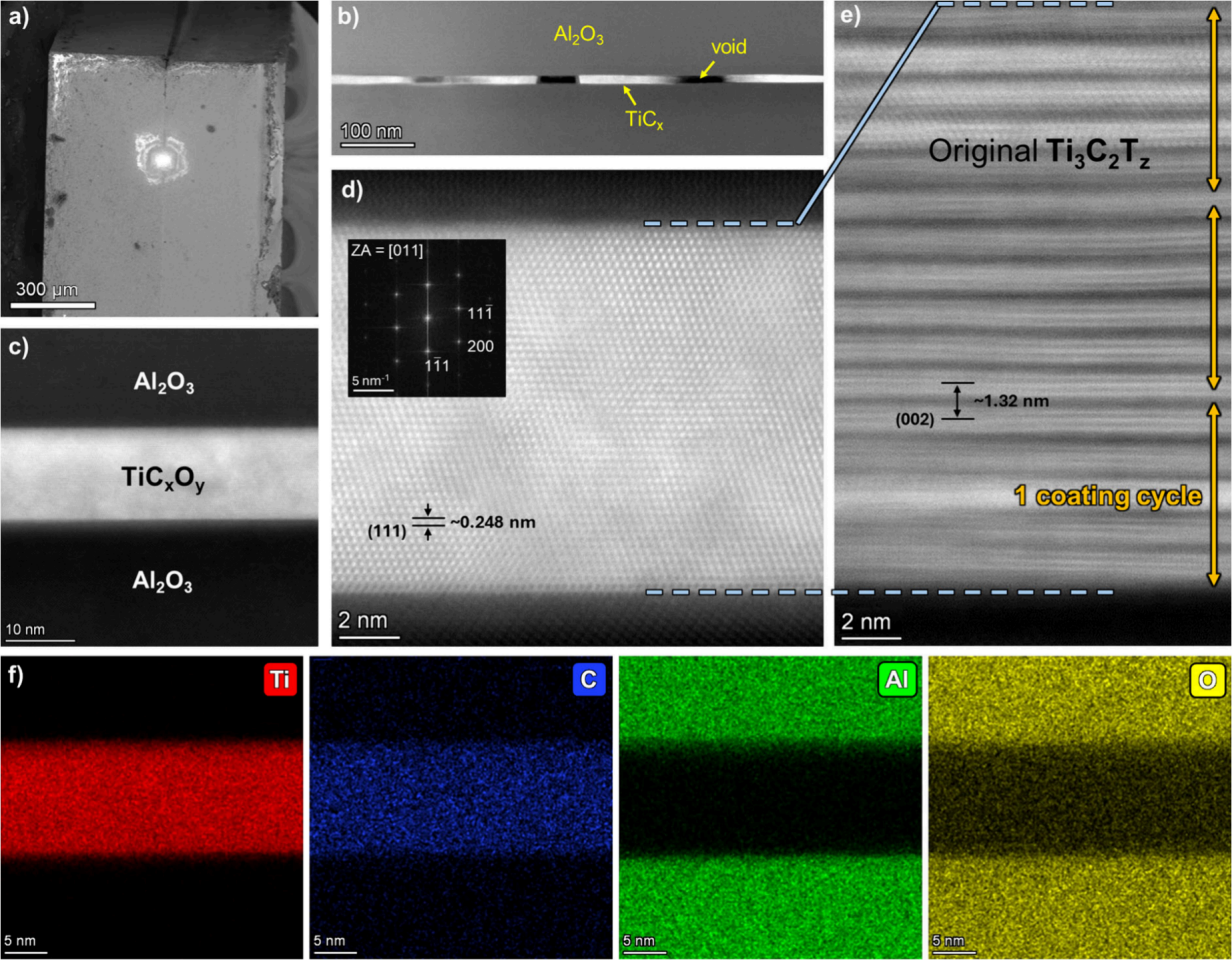
**STEM analysis of the Al**_**2**_ **O**_**3**_ **–TiC**_*x*_ **O**_*y*_ **–Al**_**2**_ **O**_**3**_ **interface cross-section.** (a) SEM image of the region of interest at the interface, marked after applying protective Pt coating during FIB preparation. (b) STEM overview image of the bonding layer cross-section, voids are visible as dark regions inside the interface. (c) High-resolution STEM image. Atomic resolution images of the resulting MX interlayer (d) after the high-temperature transformation into TiC_*x*_O_*y*_ and (e) the initial state as Ti_3_C_2_T_*z*_ MXene thin film after deposition. The *d*-spacing values were determined by FFT for the TiC_*x*_ product and from XRD analysis for the precursor MXene, as shown in Figure S2. (f) EDS elemental mapping of the sandwich interface; Ti (red), C (blue), Al (green), and O (yellow).

The presence of voids can be attributed to various factors related to the precursor MXene coating or the process by which it transforms into the MX carbide. First, there is an expected significant volume shrinkage when Ti_3_C_2_T_*z*_ transforms into TiC_*x*_ (see discussion in the next paragraph). Furthermore, despite the coatings having a relatively uniform average thickness (routinely measured by light transmission), there is always some spatial unevenness at the submicron length scale of MXene thin films^16^ – as these films are composed of dispersed discrete flakes (roughly 1.3 nm-thick, including surface terminations). Voids may form near or at thinner regions of the film. An example of a localized thickness variation of up to ∼ 50% in a relatively thick deposited MXene thin-film is shown via cross-section TEM imaging in Figure S8. Furthermore, in conjunction with thickness variations, the formation of voids upon transformation into bulk TiC_*x*_ can also be associated with coarsening mechanisms like Ostwald ripening due to the surface energy cost driving TiC to become more spherical^7^ or a layered growth mechanism that generates vacancies.^21^

The face-centered cubic (FCC) structure of the TiC_*x*_ carbide layer is evident in the high-resolution images in Figure 2c-d and the corresponding FFT inserted in Figure 2d. Although some thickness variation was observed, the typical thickness of the carbide bonding layer is approximately 12 nm, i.e. 48 atomic layers of Ti in an ABC stacking sequence. No stacking faults were observed in the TiC_*x*_. To form such a layer of presumably TiC_0.67_ stoichiometry requires a MXene thin film of roughly ∼ 20 nm thickness (consisting of 16 layers of Ti_3_C_2_T_*z*_), as shown in Figure 2e. Note that the MXene thin-film thickness would vary depending on how dry it is^22^ and the intercalation ions and termination groups, which define the interlayer *d*-spacing.^23^ After HCl treatment, the MXene (002) *d*-spacing shrank from 1.49 nm to 1.32 nm (Figure S2). Based on the initial MXene (002) *d*-spacing, relative to the resulting TiC_*x*_ (111) *d*-spacing (∼0.248 nm), the estimated theoretical shrinkage should be ∼ 43%, which agrees with the experimental STEM observations (12 nm/20 nm = 0.6). Thus, the high-temperature process leads to significant volume shrinkage upon removal of confined water, intercalants, and termination groups before/during the transformation of MXene into bulk TiC_*x*_. Note that evaporation of these elements during heating^24^ could also lead to trapped gases that contribute to void retention.

Compositional analysis with near-atomic resolution was conducted via STEM energy-dispersive X-ray spectroscopy (STEM-EDS) and atom probe tomography (APT). The quantitative elemental fraction results are presented in Table 1. STEM-EDS of a deposited MXene layer (Figure S9) revealed sizable amounts of halide terminations (∼9 at% of −F and ∼ 4 at% −Cl) as well as considerable oxygen (∼13 at%) that could be −O and −OH terminations or adsorbed organic molecules. Although it is possible for MXene to be an oxycarbide inherited from the MAX precursor,^25,26^ X-ray photoelectron spectroscopy (XPS) analysis of a deposited MXene thin-film (Figure S10) shows no detectable oxidation of Ti.^27^ Hence, it can be assumed that the oxygen observed within the deposited MXene layer is related to surface terminations alone. The XPS quantification of termination-related elements (i.e., F, Cl, and O) is in reasonably good agreement with the EDS-STEM measurements (see Table S1). Note that since the precursor MXene thin-film is deposited using single-flake colloids there should be minimal issues of unetched Al^28^ or residual AlF_3_ ^14^ as in multilayer MXene.

**Table 1:** Elemental Composition of the Ti_3_C_2_T_*z*_/TiC_*x*_O_*y*_ Layer before/after the High-Temperature Bonding Process

	Ti (at%)	C (at%)	O (at%)	F (at%)	Cl (at%)	Li (at%)
Before bonding (Ti_3_C_2_T_*z*_) [EDS]^a^	50.3 ± 4.5	23.7 ± 2.2	13.1 ± 2.7	8.9 ± 1.8	4.0 ± 0.8	-
After bonding (TiC_*x*_O_*y*_) [EDS]	59.9 ± 4.6	26.2 ± 2.5	13.9 ± 2.7	0	0	-
After bonding (TiC_*x*_O_*y*_) [APT]	51.6 ± 0.05	35.9 ± 0.05	12.2 ± 0.05	0	0	0.2 ± 0.05

a The square brackets denote the measurement technique.

The compositional analysis of the transformed MX layer by both STEM-EDS (Figure 2f) and APT (Figure S11) confirms that the −F and −Cl terminations are eliminated during the high-temperature bonding process. These halogen elements are not soluble in TiC and are expected to evaporate or dissolve into the bounding Al_2_O_3_ and diffuse away at high temperatures. On the other hand, about the same amount of oxygen found in the original MXene is still present in the transformed MX layer (Table 1). Since the TiC structure can readily accommodate TiO,^29^ it can be determined that the bulk MX layer is an oxycarbide – TiC_*x*_O_*y*_. Although such amounts of oxygen were found to exist in the carbon sublattice of various MAX phases^200^ and MXenes,^25^ the XPS analysis of the precursor MXene confirmed there was no detectable oxygen in the carbon sublattice, as discussed above. Furthermore, APT analysis of the bulk Ti_3_AlC_2_ precursor (Figure S12) revealed an oxygen content of only 1.61 ± 0.01 at. %, which is at least an order of magnitude lower than the oxygen content found in the derived Ti_3_C_2_T_*z*_ and TiC_*x*_O_*y*_. Hence, it can be determined that the higher oxygen content observed within the deposited MXene layer and MX interlayer predominantly originates from surface terminations, rather than from oxygen present in the parent MAX phase.^25,26^ We therefore postulate that the −O or −OH terminations are not removed and do not diffuse away through the Al_2_O_3_, instead they readily oxidize the MXene as it transforms into the (oxycarbide) MX phase at high temperature. Otherwise, it should have resulted in the formation of TiO_2_ ^30^ which would likely react with Al_2_O_3_ to form ternary phases such as Al_2_TiO_5_.^31^

Interestingly, APT analysis also revealed the presence of Li inside the TiC_*x*_O_*y*_ layer. APT has high elemental sensitivity and is often used to investigate light dopants^32^ that otherwise remain undetected. A 1D compositional profile across the TiC_*x*_O_*y*_–Al_2_O_3_ interface (Figure 3a,b) clearly shows that Li is found only in the oxycarbide layer. The APT mass spectra for the separate TiC_*x*_O_*y*_ and Al_2_O_3_ regions are provided in Figure S6. The origin of residual Li ions is the LiF used for etching/intercalation during the MXene synthesis process. Although Li is effectively removed by washing with HCl, some minute amounts can remain even in single-flake MXene films.^33,34^ The high temperature (1400 °C) treatment for 30 min should have allowed for thermally activated Li outward diffusion despite the limited diffusion rate of Li ions in α-Al_2_O_3_.^35^ Thus, suggesting that these minor fractions of Li ions find stable sites within the TiC_*x*_O_*y*_ lattice.

**Figure 3:**
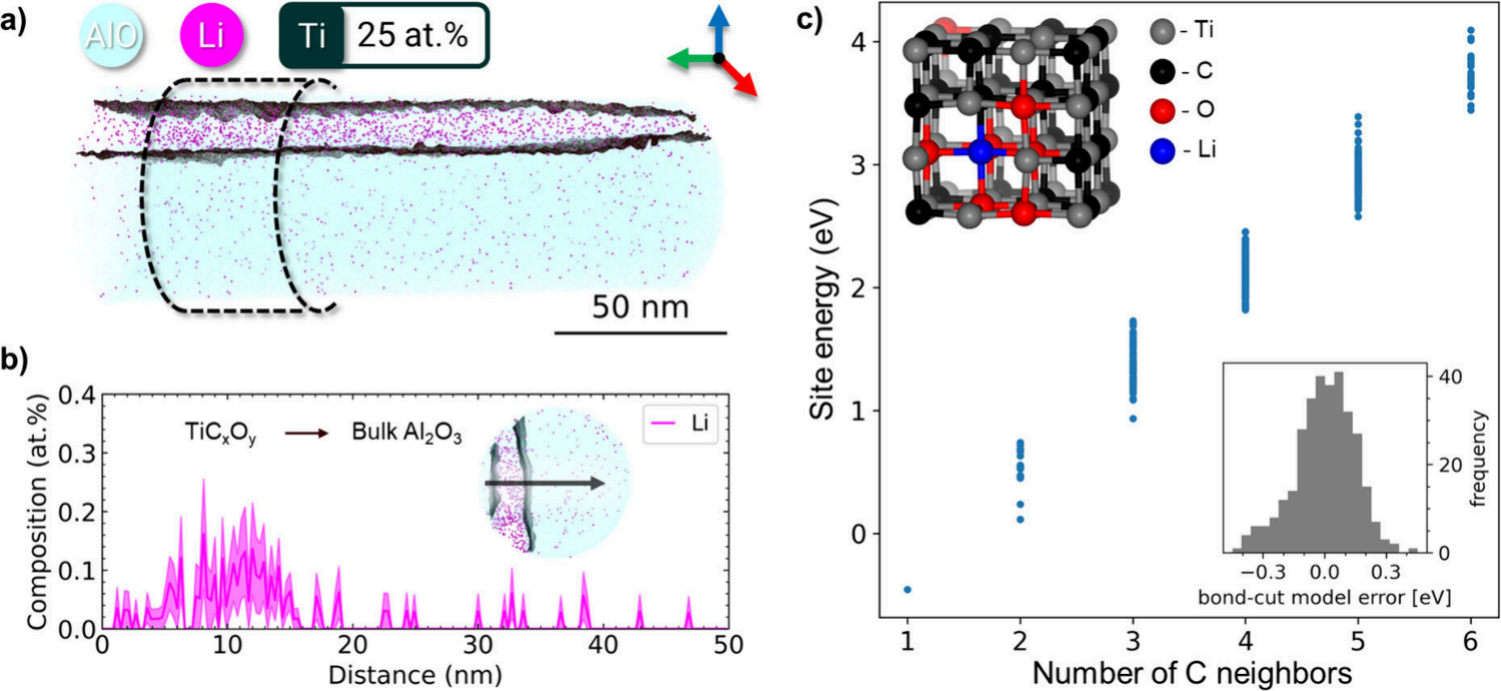
**APT analysis and DFT calculations.** (a) Reconstructed 3D atom map encompassing the TiC_*x*_O_*y*_ layer and alumina matrix. (b) 1D Li compositional profile (ø 20 nm × 50 nm) across the interface as indicated in the inset, showing the top view of the region of interest highlighted in (a). Errors are estimated according to counting statistics. (c) Li substitution energies ΔE­(_Li–Ti_) plotted against the number of C neighbors; Inserted – (top left) Ball-and-stick model of the TiC_*x*_O_*y*_ lattice with an example Li situated in the lowest energy site when neighboring 1 carbon, 4 oxygen, and 1 vacancy, (bottom right) error distribution for the bond cutting model.

To elucidate the presence and potential stabilization mechanism of Li within the TiC_*x*_O_*y*_ bonding layer, DFT calculations were performed. The detection of Li exclusively within the transformed oxycarbide region and not in the adjacent Al_2_O_3_ matrix suggests that Li atoms are incorporated into specific lattice sites that are energetically favorable under the high-temperature processing conditions. The defective structure, approximated as TiC_0.69_O_0.22_ based on the stoichiometry measured by STEM-EDS and APT, was found to be thermodynamically more stable than both stoichiometric TiC and TiO – in agreement with its formation during the transformation of Ti_3_C_2_T_*z*_. Li incorporation was assessed via two mechanisms: occupation of anion vacancies and substitution at Ti cation sites. The former pathway yielded additional energies ∼ 2.65 eV above the chemical potential of bulk antifluorite Li_2_O, indicating that Li is unlikely to occupy anion sites. In contrast, substitution of Li at Ti sites displayed a wide distribution of formation energies, ranging over more than 4 eV, which were found to correlate strongly with local chemical coordination.

A simple bond-cutting model was introduced to rationalize the energetics, revealing that sites with higher oxygen and vacancy coordination are significantly more favorable for Li substitution. Specifically, sites surrounded by four oxygen atoms, consistent with the coordination environment in LiO_2_, yielded formation energies competitive with bulk lithium oxides. Figure 3c illustrates Li substitution energies against the number of carbon neighbors and the accuracy of the bond-cutting model. Based on the statistical occurrence of such configurations in a random TiC_0.69_O_0.22_ lattice, the solubility of Li was estimated to be on the order of a few atomic percent, consistent with the experimental APT quantification. These findings suggest that Li atoms remaining from the MXene synthesis are retained during high-temperature treatment due to the availability of stable (Ti) substitutional sites in the oxycarbide lattice. The results further emphasize the critical role of termination chemistry and structural defects in defining the final composition and properties of transformed MXene-derived MX ceramics.

In summary, several key features of the Ti_3_C_2_T_*z*_-to-TiC_*x*_ transformation were uncovered by comprehensively analyzing the interface cross-section using high-precision techniques like STEM and APT. Nanovoids were found at the interface, which likely stem from the characteristics of the MXene transformation into its bulk MX counterpart. These voids could possibly be mitigated by preliminary treatment of the MXene, such as preannealing in vacuum after deposition to remove confined water.^36^ Preventing or minimizing voids could potentially improve mechanical and electrical performance of the MX nanolayers (which are expected to be slightly inferior to stoichiometric TiC^37,38^ ). Halide terminations (i.e., –F and –Cl) were effectively eliminated, whereas the oxygen terminations are suggested to have caused oxidation during the transformation into bulk TiC_*x*_, forming TiC_*x*_O_*y*_ with roughly 13 at% oxygen. It was found that having some TiO as part of the structure creates stable sites for Li (residual from the MXene synthesis) incorporation inside the oxycarbide lattice. In the case of Ti-based MXenes, the possibility of a native oxide (in other materials like SiC) that will preferably oxidize Ti should be considered. Additionally, creating thin-film TiC_*x*_ or TiC_*x*_O_*y*_ could be useful for functional applications such as separators in lithium–sulfur batteries.^39,40^ Lastly, nanolayers obtained from transforming MXenes into MX phases comprising refractory elements should be able to withstand high temperatures and be suitable as nanocomposite reinforcements, filler, or joining materials for extreme environments such as UHTCs^6,41–43^

Overall, the methodology developed herein can be applied to investigate the transformation of MXenes into their carbide/nitride MX counterparts under precise conditions. This can be used to create a model interface that mimics a grain boundary of a composite system that involves MXenes or their derived MX phases with any chosen matrix material following high-temperature processes like sintering. The controlled settings of this experimental approach enable in-depth analysis of the structure and composition to investigate reactions or phase transformations taking place in the MXene itself (or other dispersible 2D materials) as well as thermally induced interfacial reactions at multimaterial interfaces. Future studies can focus on analyzing the factors affecting MXene stability, including the number of layers, composition, termination groups (surface chemistry), temperature, pressure, and adjoining metallic or ceramic materials. For instance, whether a certain metal/ceramic will react with MXene, the temperature dependence of that reaction, and what the product phases will be. Consequently, allowing to gain fundamental insights into MXene-to-MX transformation and leveraging this knowledge for the development of novel ceramic joints, nanocomposites, and layered devices.

## Supplementary Material



## Data Availability

The data that support the findings of this study are available from the corresponding author upon reasonable request.

## Notes

Characterization of the deposited MXene thin films (Figures S1–S2, S8–S10, and Table S1), post heat treatment nanocarbide interlayer (Figures S3–S7), APT analyses (Figures S11–S13), and Methods (PDF)

## References

[1] Thakur A., Chandran BS N., Davidson K., Bedford A., Fang H., Im Y., Kanduri V., Wyatt B. C., Nemani S. K., Poliukhova V., Kumar R., Fakhraai Z., Anasori B. (2023). Step-by-Step Guide
for Synthesis and Delamination of Ti_3_C_2_T_x_ MXene. Small Methods.

[2] Jiang M., Wang D., Kim Y. H., Duan C., Talapin D. V., Zhou C. (2024). Evolution of Surface
Chemistry in Two-Dimensional MXenes: From Mixed
to Tunable Uniform Terminations. Angew. Chemie
- Int. Ed..

[3] VahidMohammadi A., Rosen J., Gogotsi Y. (2021). The World of Two-Dimensional Carbides
and Nitrides (MXenes). Science..

[4] Naguib M., Barsoum M. W., Gogotsi Y. (2021). Ten Years
of Progress in the Synthesis
and Development of MXenes. Adv. Mater..

[5] Dananjaya V., Hansika N., Marimuthu S., Chevali V., Mishra Y. K., Grace A. N., Salim N., Abeykoon C. (2025). MXenes and Its Composite
Structures: Synthesis, Properties, Applications, 3D/4D Printing, and
Artificial Intelligence; Machine Learning Integration. Prog. Mater. Sci..

[6] Wyatt B. C., Nemani S. K., Anasori B. (2021). 2D Transition Metal
Carbides (MXenes)
in Metal and Ceramic Matrix Composites. Nano
Converg..

[7] Ratzker B., Messer O., Favelukis B., Kalabukhov S., Maman N., Ezersky V., Sokol M. (2023). MXene-Based Ceramic
Nanocomposites Enabled by Pressure-Assisted Sintering. ACS Nano.

[8] Liu L., Ying G., Jiang Q., Wen D., Wang P., Wu M., Ji Z., Zheng Y., Wang X. (2024). Ultra-High-Temperature
Application of MXene: Stabilization of 2D Ti_3_C_2_T_x_ for Cross-Scale Strengthening and Toughening of 3D
TiC. J. Adv. Ceram..

[9] Nemani S. K., Gilli N., Goldy S., Kumar A., Im Y., Vorhees A. J., Wyatt B. C., Chandran BS N., Chawla N., Tucker G. J., Silvestroni L., Anasori B. (2025). Ti_3_C_2_T_x_ MXene-Zirconium
Diboride Based Ultra-High Temperature Ceramics. Adv. Sci..

[10] Yu L. P., Lu L., Zhou X. H., Xu L. (2023). Current Understanding of the Wettability
of MXenes. Adv. Mater. Interfaces.

[11] Wyatt B. C., Nemani S. K., Desai K., Kaur H., Zhang B., Anasori B. (2021). High-Temperature Stability
and Phase Transformations
of Titanium Carbide (Ti_3_C_2_T_x_) MXene. J. Phys.: Condens. Matter.

[12] Guo J., Legum B., Anasori B., Wang K., Lelyukh P., Gogotsi Y., Randall C. A. (2018). Cold Sintered
Ceramic Nanocomposites
of 2D MXene and Zinc Oxide. Adv. Mater..

[13] Dong T., Xu W., Jin M., Wu J., Mu T., Ling J., Zhou Y. (2022). A Self-Assemble Strategy
toward Conductive 2D MXene Reinforced ZrO_2_ Composites with
Sensing Performance. J. Eur. Ceram. Soc..

[14] Ratzker B., Messer O., Goldstein D., Maman N., Ezersky V., Sokol M. (2024). Challenges and Opportunities Utilizing Multilayer MXene as Precursors
for Oriented TiC_x_ in Ceramic Composites. Mater. Today Adv..

[15] Petrus M., Woźniak J., Cygan T., Lachowski A., Rozmysłowska-Wojciechowska A., Wojciechowski T., Ziemkowska W., Chlubny L., Jastrzębska A., Adamczyk-Cieślak B., Olszyna A. (2021). Silicon Carbide Nanocomposites
Reinforced with Disordered Graphitic Carbon Formed in Situ through
Oxidation of Ti_3_C_2_ MXene during Sintering. Arch. Civ. Mech. Eng..

[16] Favelukis B., Ratzker B., Miyar R., Jopp J., Upcher A., Shekhter P., Maman N., Sokol M. (2025). Without a
Grain of
Salt: Micropatterning Clean MXene Thin-Film Electronics. Nanoscale Adv..

[17] Stosz M., Narayanasamy S., Bell J., Graule T., Kata D., Blugan G. (2023). Joining of Alumina Ceramics with Ti and Zr Interlayers
by Spark Plasma Sintering. Mater. Des..

[18] Cohen S., Ratzker B., Kalabukhov S., Frage N. (2023). Diffusion Bonding of
Transparent Ceramics by Spark Plasma Sintering (SPS) Complemented
by Hot Isostatic Pressing (HIP). J. Eur. Ceram.
Soc..

[19] Zhang H. L., Xia Y., Gai J. G. (2018). Ultrathin
Active Layer for Transparent Electromagnetic
Shielding Window. ACS Omega.

[20] Engberg C. J., Zehms E. H. (1959). Thermal Expansion
of Al_2_O_3_, BeO,
MgO, B_4_C, SiC, and TiC Above 1000°C. J. Am. Ceram. Soc..

[21] Sang X., Xie Y., Yilmaz D. E., Lotfi R., Alhabeb M., Ostadhossein A., Anasori B., Sun W., Li X., Xiao K., Kent P. R. C., Van Duin A. C. T., Gogotsi Y., Unocic R. R. (2018). In Situ
Atomistic Insight into the Growth Mechanisms of Single Layer 2D Transition
Metal Carbides. Nat. Commun..

[22] Yang H., Han M., Zhang W., Yi M., Xia L., Meng F., Wang Y., Zhao S. (2024). High Performance
Mixed-Dimensional
Assembled MXene Composite Membranes for Molecular Sieving. J. Membr. Sci..

[23] Natu V., Pai R., Wilson O., Gadasu E., Badr H., Karmakar A., Magenau A. J. D., Kalra V., Barsoum M. W. (2022). Effect of Base/Nucleophile
Treatment on Interlayer Ion Intercalation, Surface Terminations, and
Osmotic Swelling of Ti_3_C_2_T_z_ MXene
Multilayers. Chem. Mater..

[24] Seredych M., Shuck C. E., Pinto D., Alhabeb M., Precetti E., Deysher G., Anasori B., Kurra N., Gogotsi Y. (2019). High-Temperature
Behavior and Surface Chemistry of Carbide MXenes Studied by Thermal
Analysis. Chem. Mater..

[25] Michałowski P.
P., Anayee M., Mathis T. S., Kozdra S., Wójcik A., Hantanasirisakul K., Jóźwik I., Piątkowska A., Możdżonek M., Malinowska A., Diduszko R., Wierzbicka E., Gogotsi Y. (2022). Oxycarbide MXenes and
MAX Phases Identification Using Monoatomic Layer-by-Layer Analysis
with Ultralow-Energy Secondary-Ion Mass Spectrometry. Nat. Nanotechnol..

[26] Persson P. O. Å., Rosén J., Mckenzie D. R., Bilek M. M. M. (2009). Formation of
the MAX -Phase Oxycarbide Ti_2_AlC_1‑x_O_x_ Studied via Electron Energy-Loss Spectroscopy and First-Principles
Calculations. Phys. Rev. B.

[27] Natu V., Benchakar M., Canaff C., Habrioux A., Célérier S., Barsoum M. W. (2021). A Critical Analysis
of the X-Ray Photoelectron Spectra
of Ti_3_C_2_T_z_ MXenes. Matter.

[28] Anayee M., Wang R., Downes M., Ippolito S., Gogotsi Y. (2025). Layer-by-Layer
Mechanism of the MAX Phase to MXene Transformation. Matter.

[29] Jiang B., Hou N., Huang S., Zhou G., Hou J., Cao Z., Zhu H. (2013). Structural Studies of TiC_1‑X_O_x_ Solid
Solution by Rietveld Refinement and First-Principles Calculations. J. Solid State Chem..

[200] Anayee M., Shekhirev M., Wang R. J., Gogotsi Y. (2024). Effect of
oxygen substitution and oxycarbide formation on oxidation of Ti3AlC2
MAX phase. J. Am. Ceram. Soc..

[30] Liu N., Li Q., Wan H., Chang L., Wang H., Fang J., Ding T., Wen Q., Zhou L., Xiao X. (2022). High-Temperature
Stability in Air of Ti_3_C_2_T_x_ MXene-Based
Composite with Extracted Bentonite. Nat. Commun..

[31] Zheng J., Hu X., Ren Z., Xue X., Chou K. (2017). Solid-State Reaction
Studies in Al2O3-TiO_2_ System by Diffusion Couple Method. ISIJ. Int..

[32] Gault B., Aota L. S., Krämer M., Kim S. H. (2025). From Impurity Ingress
to High-Performance Doping: A Perspective on Atom Probe Tomography
in Energy Materials. Scr. Mater..

[33] Krämer M., Favelukis B., El-Zoka A. A., Sokol M., Rosen B. A., Eliaz N., Kim S. H., Gault B. (2024). Near-Atomic-Scale Perspective
on the Oxidation of Ti_3_C_2_T_x_ MXenes:
Insights from Atom Probe Tomography. Adv. Mater..

[34] Krämer M., Favelukis B., Sokol M., Rosen B. A., Eliaz N., Kim S. H., Gault B. (2025). Facilitating Atom Probe Tomography
of 2D MXene Films by In Situ Sputtering. Microsc.
Microanal..

[35] Nolan A. M., Wickramaratne D., Bernstein N., Mo Y., Johannes M. D. (2021). Li+Diffusion
in Amorphous and Crystalline Al_2_O_3_ for Battery
Electrode Coatings. Chem. Mater..

[36] Fang H., Thakur A., Zahmatkeshsaredorahi A., Fang Z., Rad V., Shamsabadi A. A., Pereyra C., Soroush M., Rappe A. M., Xu X. G., Anasori B., Fakhraai Z. (2024). Stabilizing
Ti_3_C_2_T_x_ MXene Flakes in Air by Removing
Confined Water. Proc. Natl. Acad. Sci. U. S.
A..

[37] Miracle D. B., Lipsitt H. A. (1983). Mechanical Properties of Fine-Grained Substoichiomebic
Titanium Carbide. Journal of the American Ceramic
Society.

[38] Williams W. S. (1964). Scattering
of Electrons by Vacancies in Nonstoichiometric Crystals of Titanium
Carbide. Phys. Rev..

[39] Zhou T., Zhao Y., Zhou G., Lv W., Sun P., Kang F., Li B., Yang Q. H. (2017). An In-Plane
Heterostructure
of Graphene and Titanium Carbide for Efficient Polysulfide Confinement. Nano Energy.

[40] Nguyen D. L. T., Ho T. H., Nguyen T. M., Nguyen T. P., Doan T. L. L., Dang H. T., Tran M. X. (2024). Ultrathin
Titanium Carbide-Modified
Separator for High-Performance Lithium-Sulfur Batteries. Ceram. Int..

[41] Jin X., Yang J., Sun Y., Li P., Hou C., Zhao Y., Fan X. (2021). Fabrication and Characterisation
of High-Performance Joints Made of ZrB_2_-SiC Ultra-High
Temperature Ceramics. J. Eur. Ceram. Soc..

[42] Cai X. Q., Wang D. P., Qi F. G., Wang Y., Qiu Q. W., Yang Z. W. (2022). Joining of Titanium Diboride-Based Ultra High-Temperature
Ceramics to Refractory Metal Tantalum Using Diffusion Bonding Technology. J. Eur. Ceram. Soc..

[43] Wyatt B. C., Nemani S. K., Hilmas G. E., Opila E. J., Anasori B. (2024). Ultra-High
Temperature Ceramics for Extreme Environments. Nat. Rev. Mater..

