# Supplementary material for: From 2D MXenes to 3D Carbides: Transformation of Ti3C2T z Thin Films into TiC x Carbide Nanolayers
Source: Nano Lett. 2026 Apr 13;26(15):5071–7. doi: 10.1021/acs.nanolett.6c00187 (PMC13107518; doi:10.1021/acs.nanolett.6c00187)
Supplement: Supplementary file 1 [file nl6c00187_si_001.pdf]

Supporting Information

From 2D MXenes to 3D carbides: Transformation of $Ti_3C_2T_z$ thin films into TiC_x carbide nanolayers

Barak Ratzker[1,2,*], Bar Favelukis[2], Mathias Krämer[1], Christoph Freysoldt[1], Yiftach Kushnir[2], Asaf Nitsan[2], Alexander Upcher[3], Nitzan Maman[3], Or Messer[2], Dierk Raabe[1], Baptiste Gault[1,4], Maxim Sokol[2,*]

[1] *Max Planck Institute for Sustainable Materials, Max-Planck-Str. 1, 40237 Düsseldorf, Germany*

[2] *Department of Materials Science and Engineering, Tel Aviv University, P.O.B 39040, Ramat Aviv 6997801, Israel*

[3] *Ilse Katz Institute for Nanoscale Science and Technology, Ben-Gurion University of the Negev, P.O.B 653, Beer-Sheva 8410501, Israel*

[4] *Department of Materials, Imperial College London, London, SW7 2AZ, UK*

* Corresponding authors: b.ratzker@mpi-susmat.de; sokolmax@tauex.tau.ac.il

Table of contents:

Figure S1 – XRD patterns of MAX and MXene and SEM image of deposited film……..…..2

Figure S2 – XRD patterns and d-spacing of MXene before and after HCl treatment…...…....2

Figure S3 – In-line transmittance of polished bonded Sapphire–TiC_xO_y–Sapphire…….…….3

Figure S4 – STEM images of voids at Sapphire–TiC_xO_y–Sapphire interfaces.…..…………...3

Figure S5 – STEM images of the further Sapphire–TiC_xO_y–Sapphire interfaces………..…..3

Figure S6 – STEM images showcasing reaction with Al_2O_3 at the interfaces..……………….4

Figure S7 – STEM image and FFTs of Sapphire and TiC_xO_y……………….……………….4

Figure S8 – STEM image of deposited MXene thin film……………………………..……..4

Figure S9 – STEM-EDS analysis of deposited MXene thin film……………………..……..5

Figure S10 – XPS analysis of deposited MXene thin film…………………………..……..…5

Table S1 – XPS quantitative results of deposited MXene thin film………………..…..……..5

Figure S11 – APT analysis of the Al_2O_3–TiC_xO_y interface…………………………….……6

Figure S12 – APT analysis of MAX phase precursor…….………………….……….………7

Figure S13 – Correlation histogram for carbon multiple events……………………….………7

Methods……………………………………………………………………….……….…….…….8

References……………………………………………………………..………………………11

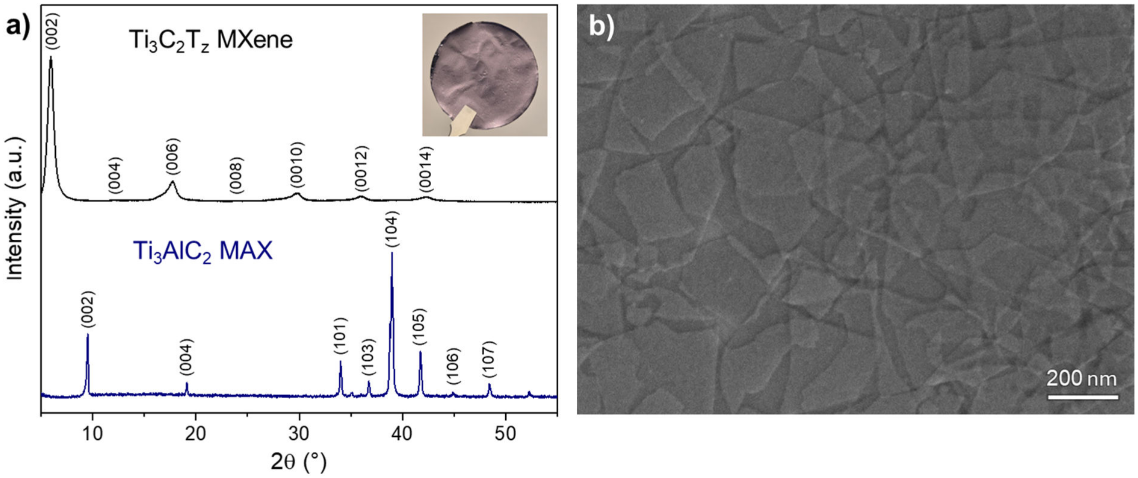

Figure S1. (a) XRD patterns of the MAX phase precursor and the derived single-flake MXene freestanding film (inset shows photograph of a film). (b) High-resolution SEM image of deposited MXene thin film (1 deposition cycle).

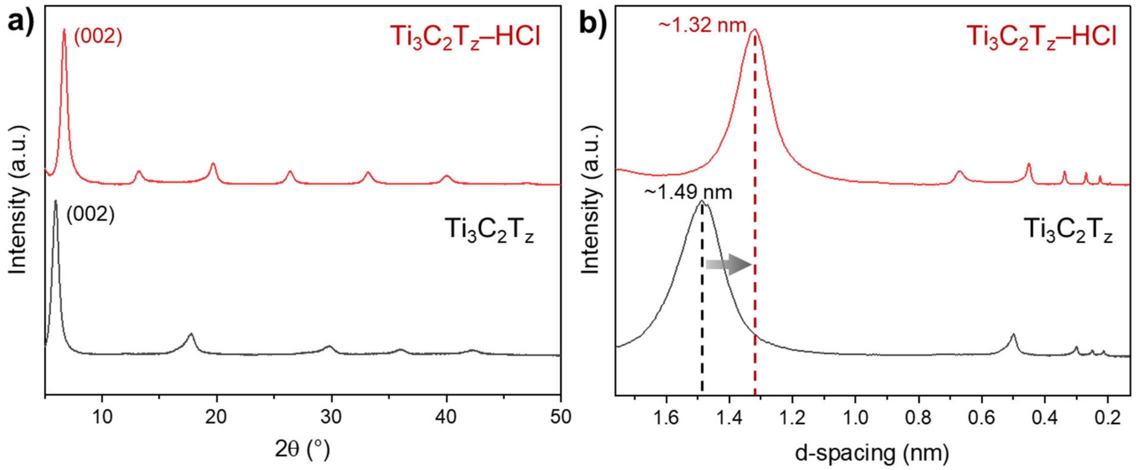

Figure S2. (a) XRD patterns of the MXene before and after HCl treatment. (b) The corresponding peak shift and decrease in the (002) d-spacing due to the removal of intercalated Li ions.

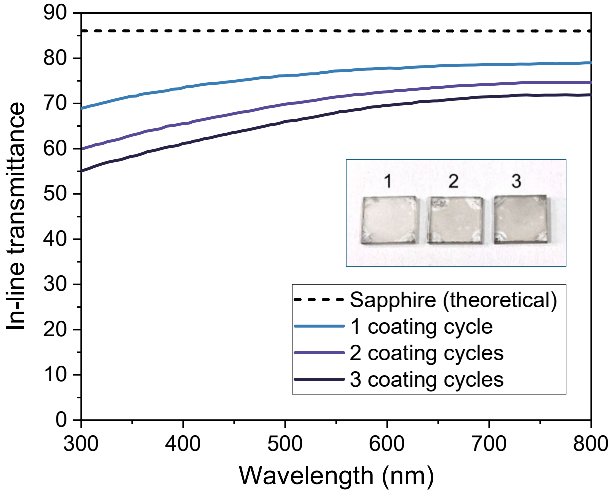

Figure S3. (a) In-line transmittance of the polished bonded sapphire samples; with the carbide interlayer consisting of 1, 2, or 3 spin-coating cycles of MXenes corresponding to a thin-film thickness of roughly ~6.5, ~13, and ~20 nm. A photograph of the polished, bonded samples is inserted.

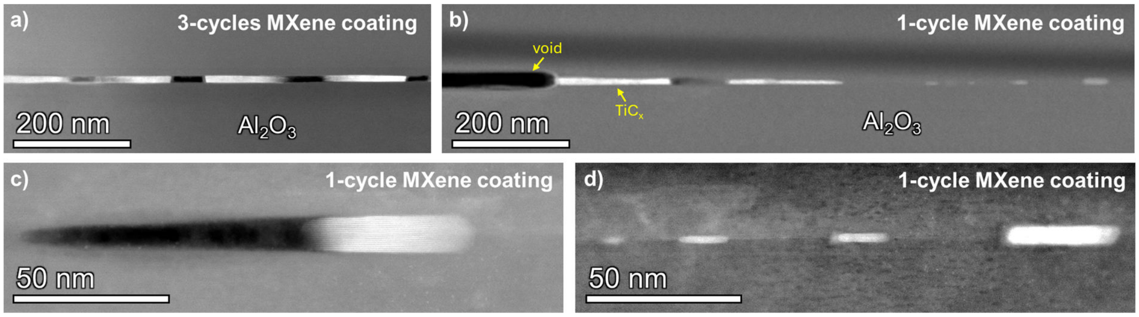

Figure S4. STEM images of the interfaces in bonded sapphire dies after SPS with deposited MXene-derived TiC_x in between; (a) 3 spin-coating cycles and (b)-(d) 1 spin-coating cycle. Note the higher uniformity of the bonding layer achieved for the thicker MXene-thin film.

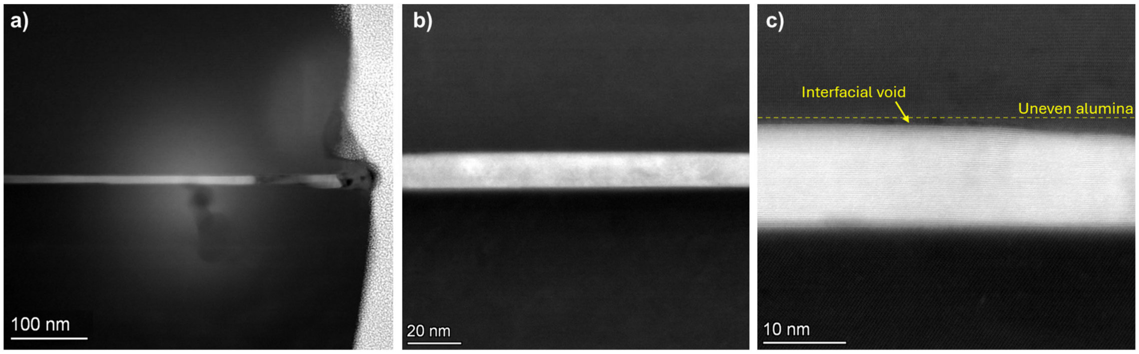

Figure S5. (a) an interface region of hundreds of microns without noticeable voids. (b) Zoom in on part of the region shown in (a). (c) a region showing that the Al_2O_3 single crystal has deformed (by creep) and showcases an uneven surface at the upper part of the interface.

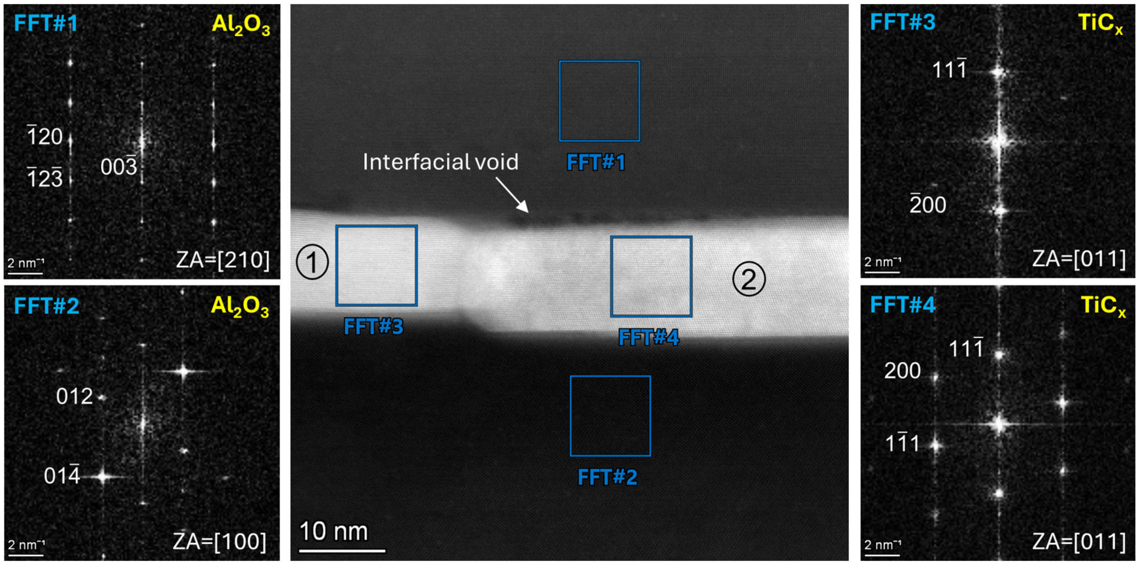

Figure S6. High resolution STEM of two TiC_x nanograins in the interface with corresponding FFTs (labeled #1–4) of the Al_2O_3 dies (which were placed so they were oriented 90° to each other) and TiC_x where grain 1 is not completely in zone axis as the Al_2O_3 and grain 2. An interfacial void was observed between part of the TiC_x and Al_2O_3.

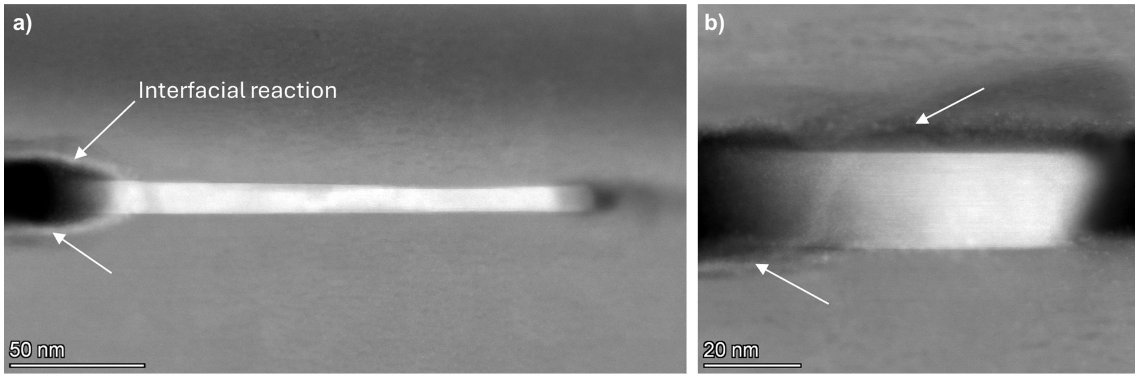

Figure S7. STEM images of regions in the interface for the 1-cycle deposited MXene after SPS, showing evidence of reaction with the alumina at (a) void regions and (b) between TiC_x and Al_2O_3. This may be associated with local high concentrations of impurities such as LiF that were not fully removed.

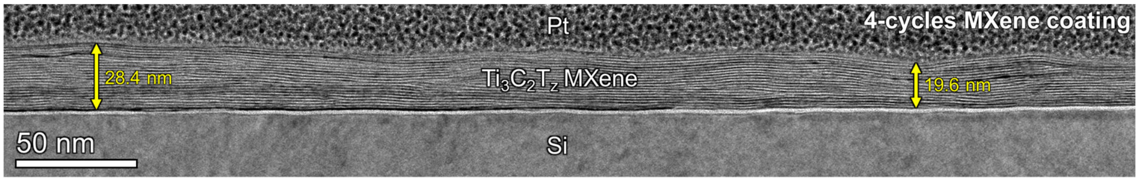

Figure S8. High-resolution TEM image showcasing a ~0.5 µm span cross-sectional view of a 4-cycle spin-coated MXene thin-film deposited on a Si die. On average the thickness is relatively uniform and conforming to the expected thickness (~26 nm) but there are evident local variations in thickness of up to nearly 50% (i.e., minima and maxima of 19.6 and 28.4 nm).

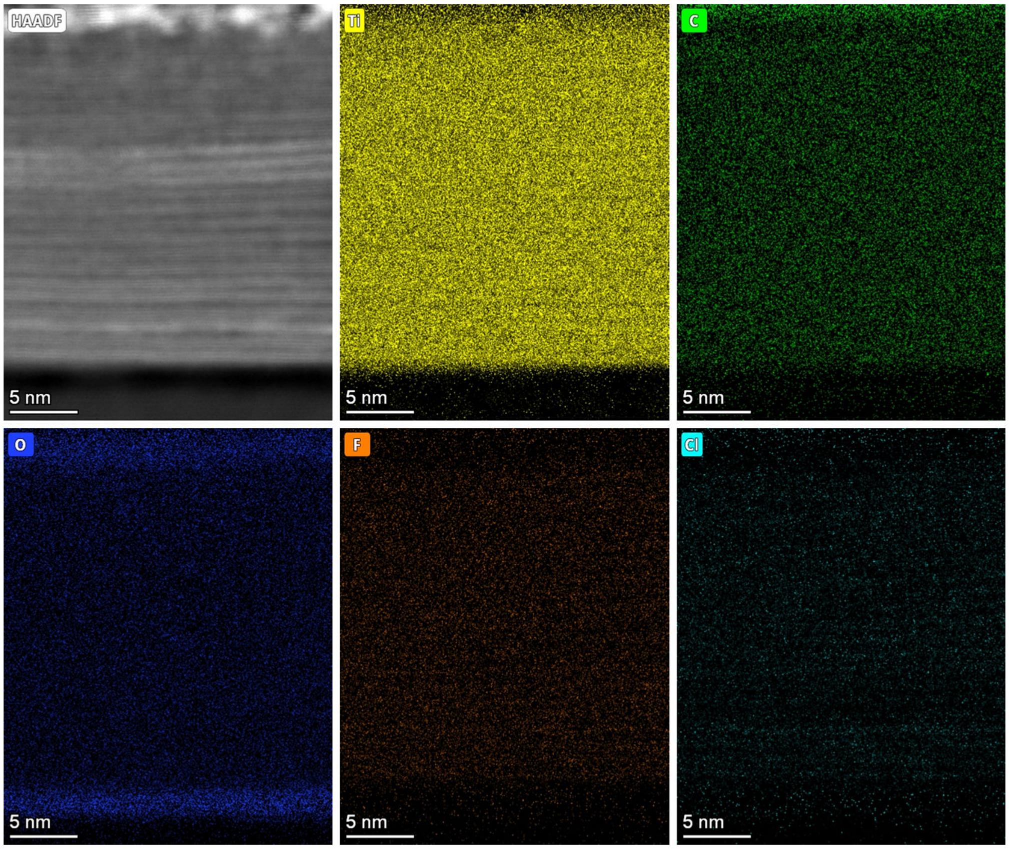

Figure S9. STEM-EDS analysis of a representative MXene thin-film deposited on a Si die (four spin-coating cycles). Note the presence of halogen elements in the deposited MXene prior to thermal treatment.

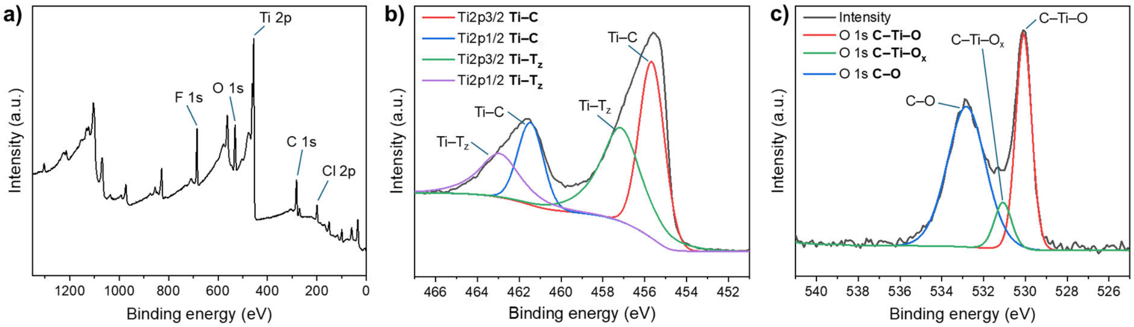

Figure S10. (a) XPS spectra of the analyzed MXene thin-film deposited by spin-coating and deconvoluted peaks for (b) Ti p1/2 and Ti p3/2 and (c) O 1s. The binding energies detected for Ti and O suggest that the oxygen present is associated with terminations (or adsorbed organics) and there is no apparent oxidation of Ti.

Table. S1. XPS surface elemental quantification of a deposited MXene thin film.

	Ti (at%)	C (at%)	O (at%)	F (at%)	Cl (at%)
MXene surface composition	31.1	30.7	18.5	14.7	4.9

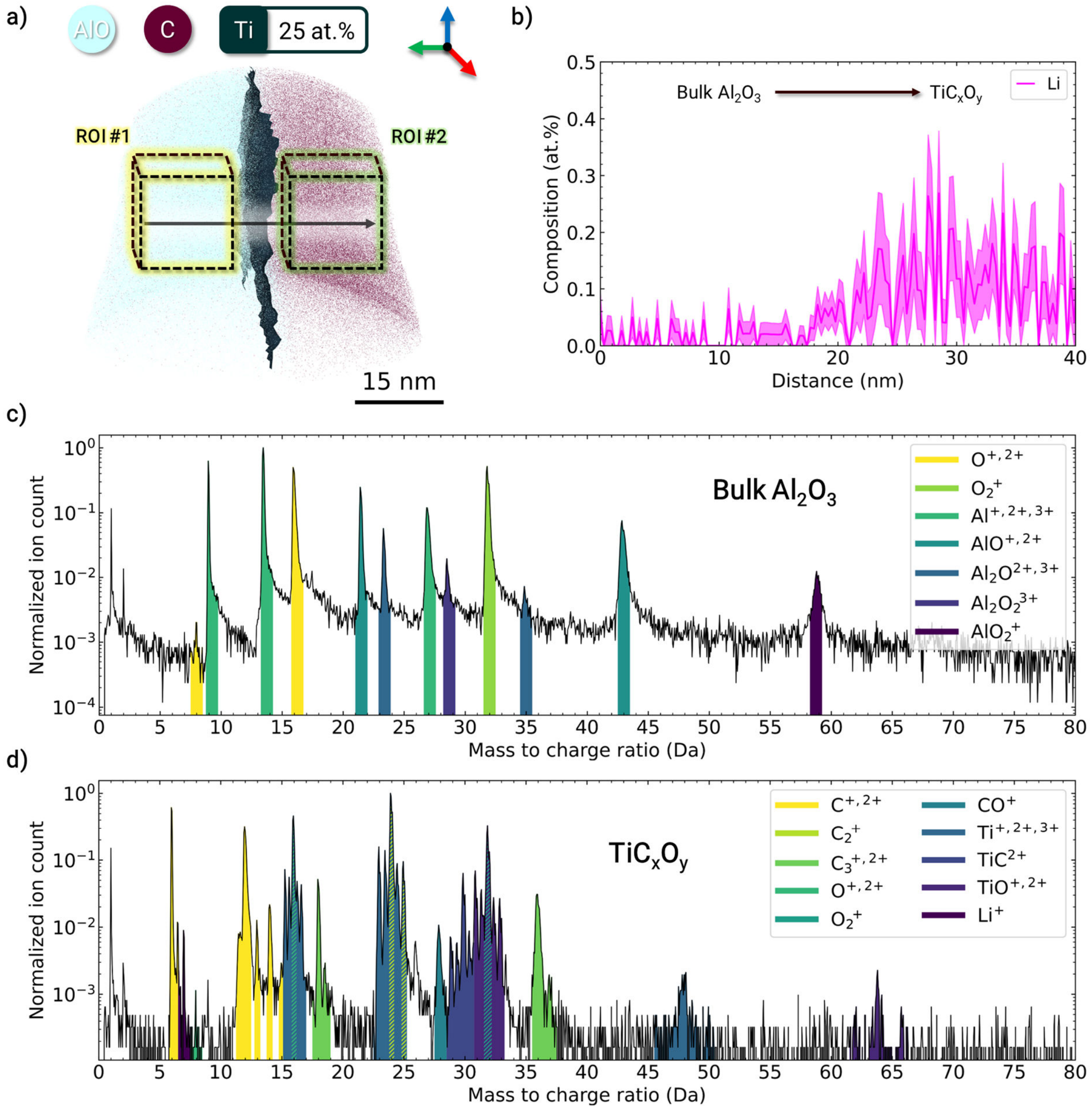

Figure S11. APT analysis of the Al_2O_3–TiC_xO_y interface. (a) Reconstructed 3D atom map. (b) 1D Li compositional profile (ø 20 nm x 40 nm) across the interface as indicated in (a). Errors are estimated according to counting statistics. (c) Mass spectra of the cubic (15 nm x 15 nm x 15 nm) region of interest #1 containing the bulk Al_2O_3 in (a). (d) Mass spectra of the cubic (15 nm x 15 nm x 15 nm) region of interest #2 containing the bulk TiC_xO_y in (a). Bin width for both mass spectra is 0.05 Da.

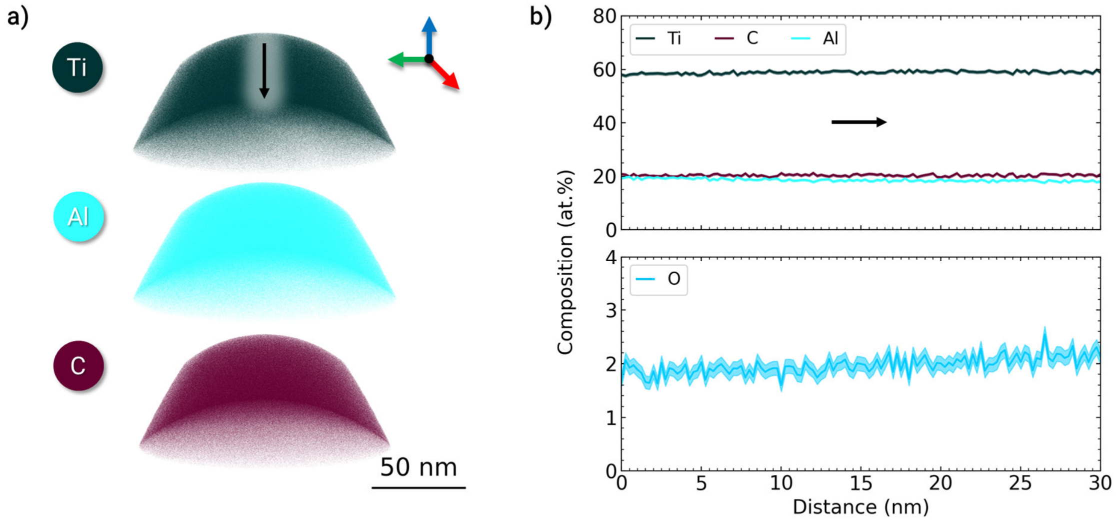

Figure S12. Analysis of the Ti_3AlC_2 MAX phase precursor. (a) Reconstructed 3D atom maps. (b) 1D compositional profile (ø 30 nm x 30 nm) as indicated in (a). Oxygen was detected in the material at (1.61 ± 0.01) at.%. Carbon was quantified at (19.73 ± 0.02) at.%, although this value is likely underestimated due to the complex evaporation behavior of carbon, which frequently results in multiple detection events (i.e., more than one ion detected per pulse) and can lead to compositional biases.

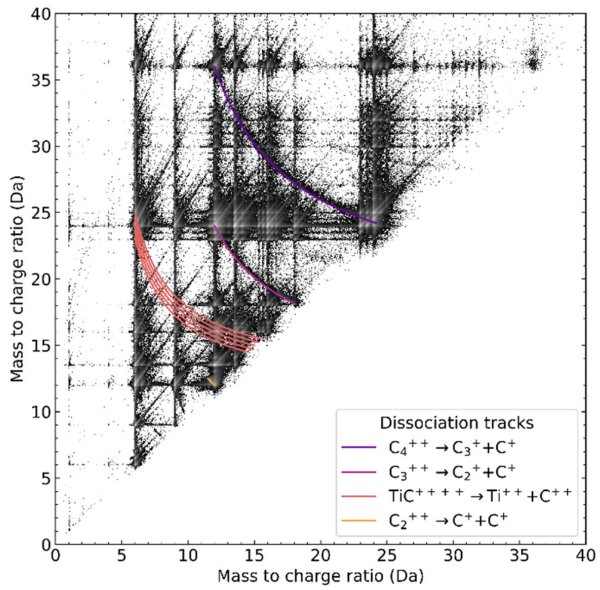

Figure S13. Correlation histogram enabling analysis of multiple events in the APT dataset of the Ti_3AlC_2 MAX phase, with identified dissociation tracks. The correlation histogram plots the mass to charge ratios of two ions from the same multiple detection event against each other. The dissociation of field evaporated molecular ions can result in characteristic dissociation tracks in the histogram if the dissociation is accompanied by a kinetic energy release. During their flight to the detector, carbon-containing molecular ions may dissociate into fragments with mass to charge ratios different from that of the parent ion, causing them to lose correlation with the pulse and potentially be lost in the background. For the $C^{2++} \rightarrow C^{+} + C^{+}$ dissociation track, the detector's dead time may cause only one of the carbon ions to be registered[1]. Interested readers are referred to the works by Saxey[2] and Peng et al.[3] for further information on the analysis of correlation histograms.

Methods

MAX phase synthesis

Titanium carbide (TiC, 99.5%, Alfa Aesar), titanium (Ti, 99.7%, Strem), and aluminum (Al, 99.7%, Strem) powders were combined in a molar ratio of 2:1:1.1 and mixed using a tumbler mixer with stainless steel balls at 200 rpm for 18 h. The resulting powder blend was pressed into a pellet at room temperature under a uniaxial pressure of 5 MPa. This pellet was then heated in a tube furnace under an argon atmosphere at 1450 °C for 3 h, with both heating and cooling rates set to 5 °C/min. The synthesized Ti_3AlC_2 MAX phase was subsequently ground and sieved to obtain a fine powder with particles smaller than 20 μm.

MXene synthesis

$Ti_3C_2T_z$ MXene was synthesized by etching and exfoliation of the precursor Ti_3AlC_2 powder. Briefly, 1 g of MAX phase was gradually added to 20 ml of 10.2 M HCl (32%, Bio-Lab) containing 1.6 g of lithium fluoride (LiF, 99%, Strem) at 45 °C, and the mixture was stirred for 24 h. After etching, the product was washed by repeated centrifugation at 3500 rpm with deionized water until the supernatant reached a pH of ~6. To achieve exfoliation, the resulting sediment was redispersed in DI water and sonicated in a cooled ultrasonic bath for 30 min (maintained at 5 °C). The suspension was then centrifuged at 3500 rpm for 30 min to remove unexfoliated material. The supernatant, containing an approximate concentration of 12 g/L delaminated $Ti_3C_2T_z$, was purged with nitrogen gas for 15 min and stored at 4 °C.

MXene thin-film deposition

MXene deposition was carried out following a similar protocol to that described in our previous study[4]. Square 8×8 mm^2 and 400 nm thick sapphire dies cut from a 2” wafer (c-plane (0001), EPI-ready; Semiconductor Wafer Inc., SWI) were treated with piranha solution (1:3 H_2O_2/ H_2SO_4; H_2SO_4 98%, SDFCL; H_2O_2 35%, ThermoFisher Scientific) for 15 min to enhance surface hydrophilicity. The dies were then spin-coated with a 7 g/L MXene dispersion at 2000 rpm for 1 min, followed by a 30 s heat treatment at 80 °C on a hot plate, and finally spin-cleaning using 0.5 M HCl at 2000 rpm for 1 min. Each deposition cycle produced a ~6 nm-thick MXene film, and 1 to 3 deposition cycles were applied per sample.

SPS bonding process

The excess MXene at the die corners were scraped off using plastic-ended tweezers and each MXene-coated die was then covered by an additional sapphire die (polished side face down on the MXene layer). The prepared sapphire–MXene–sapphire die "sandwiches" were arranged together in a 30-mm graphite die using a ~0.4 mm-thick graphite foil with cutouts to make sure the dies stay in place (Figure 1d). Thin 0.15 mm-thick graphite foils were used to outline the interior of the graphite die (mold). Graphite felt was placed around the mold for thermal insulation. The SPS procedure included applying a pressure of 18 MPa and heating up with a heating rate of 25 °C/min to 1400 °C, holding for 30 min, and cooling at a rate of 25 °C/min and alleviating the pressure at the end of the holding stage. The temperature was monitored by means of a pyrometer measuring the temperature through a hole in the upper punch, at a distance of ~5 mm away from the samples.

In-line transmittance measurements

The bonded sandwich structures were polished on both sides, using grinding SiC, diamond slurry, and oxide nanoparticles suspension (OPS). The in-line transmittance was then measured at a wavelength of 300–800 nm using a UV-vis spectrophotometer (Evolution 220, Thermo Scientific).

FIB sample preparation

Prior to any FIB preparation, the samples were cut using a dicer into smaller squares (four 4x4 mm^2) to allow direct access to the interface at the central part of the bonded sandwich structure. **TEM lamellae specimens** of the interface cross-sections were prepared using a dual-beam FIB (FEI Helios G4 UC). Prior to lift-out the interface region of interest was coated by Pt electron deposition followed by W ion deposition in order to protect the surface and enable clean cutting with minimal curtaining. **Needle-shaped APT specimens** of the interface were prepared using a dual-beam Xe-plasma FIB (FEI Helios PFIB) and a dual-beam Ga FIB (FEI Helios Nanolab 600), following a common lift-out and sharpening protocol[5]. The Xe-plasma FIB was chosen for the lift-out due to higher milling rates for sapphire, while the Ga FIB was used for sharpening due to the lower beam spread and therefore better control over the specimen shape. The MAX phase APT specimens were prepared using only the dual-beam Ga FIB.

STEM analysis

Examination of the interface by STEM was conducted using a Spectra 200 (Thermo Fisher Scientific) equipped with a probe Cs-corrector (S-CORR) and cold-FEG electron source at an accelerating voltage of 200 kV. The images were recorded in STEM mode with high angle annular dark field (HAADF) detector with a semi-convergence angle of 30 mrad, a probe current of 250 pA, and an inner collection angle of 56.0 mrad. The EDS spectra were collected with a Super-X (Thermo Fisher Scientific) four-segment silicon drift detector (SDD). The images and EDS spectra were processed with Thermo Fisher Scientific Velox software (version 3.15).

APT analysis

APT specimens were analyzed using a 5000XS (straight flight path) local electrode atom probe (Cameca Instruments), operating in ultraviolet (λ = 355 nm) laser-pulsing mode. For the analysis of the interface, parameters were set to a base temperature of 60 K, a laser pulse energy of 40 pJ, a laser pulsing rate of 125 kHz, and a target detection rate 1 ions per 100 pulses on average. The MAX phase was analyzed using a base temperature of 50 K, a laser pulse energy of 30 pJ, a laser pulsing rate of 250 kHz, and a target detection rate 1 ions per 100 pulses on average. Data reconstruction and analysis was done with the commercial software AP Suite 6.3 (Cameca Instruments) following the default voltage-based reconstruction algorithm.

DFT calculations

First-principles calculations (DFT) were carried out within the plane-wave projector augmented wave (PAW) formalism[6], as implemented in the SPHInX code[7], using the PBE exchange-correlation functional[8] and PAW setups from VASP[9]. To model the experimentally observed oxycarbide composition, defective $Ti_{32}C_{22}O_7$ rocksalt supercells ($TiC_{0.69}O_{0.22}$) were constructed, where carbon, oxygen, and three anion vacancies were randomly distributed over 32 available anion sites. A total of 100 independent configurations were generated. Internal coordinates were optimized for each configuration individually, while the lattice constant was taken as the average across the ensemble.

Chemical potentials of C and O were estimated based on site addition energies evaluated over 35 configurations (105 sites per element). Although the energy distributions were unimodal, they exhibited non-Gaussian behavior with standard deviations of 0.38 eV (C) and 0.22 eV (O).

These values were used to approximate the mean chemical potentials for further thermodynamic analysis. The titanium chemical potential was then derived via:

$$\mu(Ti) = 1/32\ [E_{avg}(Ti_{32}C_{22}O_7) - 22\ \mu(C) - 7\ \mu(O)] \quad (1)$$

Li incorporation was studied in two ways. First, Li atoms were inserted into the available anion vacancies across all 100 bulk configurations (300 sites). These configurations yielded addition energies ~2.65 eV above the chemical potential (2) of bulk anti-fluorite Li_2O, indicating that Li incorporation via this pathway is thermodynamically unfavorable.

$$\mu_{ref}(Li) = 1/2\ [E(Li_2O) - \mu(O)] \quad (2)$$

Second, Li substitution at Ti cation sites (3) was evaluated for all 32 cation positions across 10 configurations (320 cases).

$$\Delta E(Li_{Ti}) = E(Ti_{31}C_{22}O_7Li) - E(Ti_{32}C_{22}O_7) - \mu_{ref}(Li) + \mu(Ti) \quad (3)$$

The computed formation energies showed a wide spread (>4 eV), with a clear correlation to the local coordination environment. A bond-counting model was used to quantify the relationship:

$$\Delta E(Li_{Ti}) \approx \Delta E_C\ n(C) + \Delta E_O\ n(O) + \Delta E_V\ n(V) \quad (4)$$

where , n(C), n(O), and n(V) represent the number of neighboring carbon, oxygen, and vacancy sites, respectively. The model provided excellent agreement with DFT data (standard deviation of 0.14 eV). Sites with four oxygen neighbors were found to be energetically competitive with bulk LiO_2, aligning with favorable Li environments. Based on the statistical frequency of such sites, the solubility of Li in $TiC_{0.69}O_{0.22}$ was estimated to be approximately 5 at%.

References

(1) Peng, Z.; Vurpillot, F.; Choi, P.; Li, Y.; Raabe, D. On the Detection of Multiple Events in Atom Probe Tomography. *Ultramicroscopy* **2018**, *189*, 54–60. https://doi.org/10.1016/j.ultramic.2018.03.018.

(2) Saxey, D. W. Correlated Ion Analysis and the Interpretation of Atom Probe Mass Spectra. *Ultramicroscopy* **2011**, *111* (6), 473–479. https://doi.org/10.1016/j.ultramic.2010.11.021.

(3) Peng, Z.; Zanuttini, D.; Gervais, B.; Jacquet, E.; Blum, I.; Choi, P.; Raabe, D.; Vurpillot, F.; Gault, B. Unraveling the Metastability of C_n^{2+} (n =2−4) Clusters. *J. Phys. Chem. Lett.* **2019**, *10*, 581−588. https://doi.org/10.1021/acs.jpclett.8b03449.

(4) Favelukis, B.; Ratzker, B.; Miyar, R.; Jopp, J.; Upcher, A.; Shekhter, P.; Maman, N.; Sokol, M. Without a Grain of Salt: Micropatterning Clean MXene Thin-Film Electronics. *Nanoscale Adv.* **2025**, *7*, 2329–2337. https://doi.org/10.1039/d4na00983e.

(5) Kantor, M. Y.; Donné, A. J. H.; Jaspers, R.; Van Der Meiden, H. J. Thomson Scattering System on the TEXTOR Tokamak Using a Multi-Pass Laser Beam Configuration. *Plasma Phys. Control. Fusion* **2009**, *51* (5), 055002. https://doi.org/10.1088/0741-3335/51/5/055002.

(6) Blöchl, P. E. Projector Augmented-Wave Method. *Phys. Rev. B* **1994**, *50* (24), 17953. https://doi.org/10.1103/PhysRevB.50.17953.

(7) Boeck, S.; Freysoldt, C.; Dick, A.; Ismer, L.; Neugebauer, J. The Object-Oriented DFT Program Library S / PHI / NX. *Comput. Phys. Commun.* **2011**, *182* (3), 543–554. https://doi.org/10.1016/j.cpc.2010.09.016.

(8) Perdew, J. P.; Burke, K.; Ernzerhof, M. Generalized Gradient Approximation Made Simple. *Phys. Rev. Lett.* **1996**, *77* (18), 3865–3868. https://doi.org/10.1103/PhysRevLett.77.3865.

(9) Kresse, G.; Joubert, D. From Ultrasoft Pseudopotentials to the Projector Augmented-Wave Method. *Phys. Rev. B* **1999**, *59* (3), 1758. https://doi.org/10.1103/PhysRevB.59.1758.
